# Synthesis, Evaluation of Anticancer Activity and QSAR Study of Heterocyclic Esters of Caffeic Acid 

**Published:** 2013

**Authors:** Shima Hajmohamad Ebrahim Ketabforoosh, Mohsen Amini, Mohsen Vosooghi, Abbas Shafiee, Ebrahim Azizi, Farzad Kobarfard

**Affiliations:** a *Department of Medicinal Chemistry, Faculty of Pharmacy and Pharmaceutical Sciences Research Center, Tehran University of Medical Sciences, Tehran 14176, Iran. *; b *Department of Medicinal Chemistry, Faculty of Pharmacy and Drug Design and Development Research Center, Tehran University of Medical Sciences, Tehran 14176, Iran.*; c *Molecular Research Laboratory, Department of Pharmacology and Toxicology, Faculty of Pharmacy, Medical Sciences, University of Tehran, Tehran, Iran. *; d *Department of Medicinal Chemistry, School of Pharmacy, and Phytochemistry Research Center, Shahid Beheshti University of Medical Sciences, Tehran, Iran. *

**Keywords:** Caffeic acid, Mitsunobu, Electrooxidation, Cytotoxic activity

## Abstract

Caffeic acid phenethyl ester (CAPE) suppresses the growth of transformed cells such as human breast cancer cells, hepatocarcinoma , myeloid leukemia, colorectal cancer cells, fibrosarcoma, glioma and melanoma. A group of heterocyclic esters of caffeic acid was synthesized using Mitsunobu reaction and the esters were subjected to further structural modification by electrooxidation of the catechol ring of caffeic acid esters in the presence of sodium benzenesulfinate and sodium toluensulfinate as nucleophiles. Both heterocyclic esters of caffeic acid and their arylsulfonyl derivatives were evaluated for their cytotoxic activity against HeLa, SK-OV-3, and HT-29 cancer cell lines.

HeLa cells showed the highest sensitivity to the compounds and heterocyclic esters with no substituent on catechol ring showed better activity compared to their substituted counterparts. QSAR studies reemphasized the importance of molecular shape of the compounds for their cytotoxic activity.

## Introduction

Cancer is one of the most important causes of mortality worldwide. Although there are many treatment options for this disease such as radiotherapy, surgical operation, immunotherapy and chemotherapy, cancer still remains a serious clinical problem (1). Accordingly we need new approach for cancer therapy. Among the wide spectrum of cancer treatment strategies, chemotherapy has significant role in management of cancer. Therefore discovery of novel anti cancer agents is one of the necessities in cancer research. The main objectives of anti cancer drug development are decreasing the toxicity and maximizing the efficacy. 

**Figure 1:**
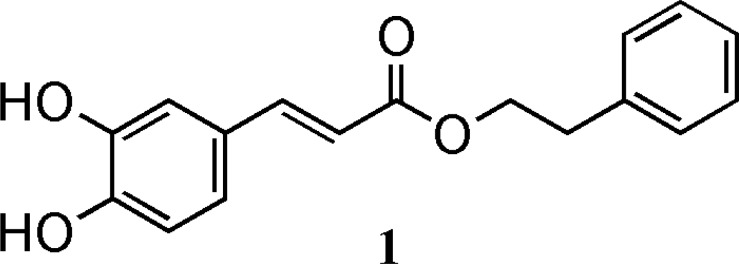
Structure of Caffeic acid phenethyl ester

Caffeic acid (CA) and caffeic acid phenethyl ester (CAPE) (Figure 1) have been identified as biologically significant components of honey bee propolis (2). Existing reports indicate that CAPE has various biological properties including antibacterial, antiviral, anti-inflammatory, antioxidant, anti-HIV, anti thrombosis, and tumor cell arrest (3-5). CAPE suppresses the growth of transformed cells such as human breast cancer cells, hepatocarcinoma , myeloid leukemia, colorectal cancer cells, fibrosarcoma, glioma and melanoma (6-11). As a result CAPE is an appropriate lead compound for anti cancer studies.

Structure-activity relationship studies for caffeic acid derivatives suggest that catechol ring is essential for cytotoxicity of CAPE and it has been proven that part of its anticancer effect is due to antioxidant mechanism of catechol ring. Moreover metylation of hydroxyl groups reduces the activity of CAPE. Conjugated double bond is also required for inhibition of cell growth (12). 

Aliphatic esters of caffeic acid with 2, 5- or 3, 5- or 2, 3- dihydroxy benzene rings had comparable cytotoxicity to doxorubicin. Replacement of the ester by amide or hydroxy groups substantially decreased or abolished cytotoxicity (13). QSAR studies on aliphatic esters of CA showed the dependence of cytotoxicity of this compound on hydrophobicity and steric factors (14). Based on the previous studies on CAPE analogues, it has been discovered that replacing phenyl with cyclohexyl increases the cytotoxic activity. Elongation of alkyl chain of phenethyl up to eight carbons, increases strength of compound. Furthermore in aliphatic esters, elongation of the chain up to 17 carbons enhances potency (15).

Despite the interesting trends observed in SAR studies of CAPE, only few analogues of this compound have been synthesized and studied mostly because of the surprisingly difficult synthesis of these groups of compounds.

The first part of this study was aimed at the synthesis of heterocyclic esters of caffeic acid and evaluation of their cytotoxic activity in compared to that of caffeic acid phenethyl ester (CAPE). 

Phenolic carboxylic acids or alcohols are notorious for their unamenability to most of methods of esterification. The tough reaction conditions which is needed in Fischer estrification method (strong protic acid) along with the need to use excess alcohol makes this strategy of limited applicability for making caffeic acid esters.

Lewis acid-catalyzed acylations will not solve the problem either (16). Using acylhalides to make esters via acyl nucleophilic substitution requires protection of phenolic hydroxyl groups since carboxylic acids will not discriminate between aliphatic hydroxyl (alcoholic) and aromatic hydroxyl (phenolic) groups. 

Stuwe *et al. *reported a method based on the reaction of cesium salt of phenolic carboxylic acid with proper alkyl halide to give the desired ester but unsatisfactory yield and the need for excess of halide are still major issues for this method (17).

The enzymatic and ultrasound-accelerated enzymatic synthesis of caffeic acid phenethyl ester from caffeic acid and phenethyl alcohol have been also investigated but these methods have not gone beyond simple alkyl esters and phenethyl esters (18-19).

Knoevenagel reaction has also been used as an indirect method for the synthesis of caffeic acid 3, 4-dihydroxyphenethyl ester (20). The major drawback for this method is unavailability of the atypical starting materials which are required to implement the method.

Perhaps the most successful method in phenolic ester synthesis is the report by Appendino *et al. *in which Mitsunobu reaction has been employed to make a few polyphenolic esters (21).

**Figure 2:**
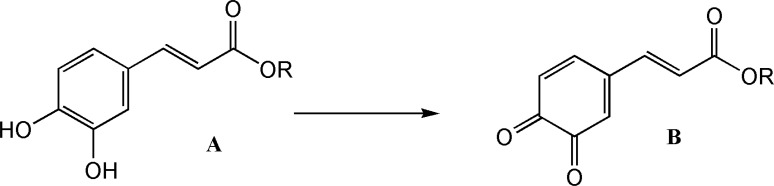
Oxidation of catechol ring

CAPE and its analogues have a diphenolic ring (catechol) in their structures with could be electrochemically converted to 1, 2-benzoquinone by a reversible two-electron oxidation (Figure 2).

The electrochemically generated *o*-benzoquinones are quite reactive intermediate which in a proper condition can be attacked by a variety of nucleophiles and undergo various reactions such as Micheal addition. Sodium benzenesulfinate and Sodium toluene-4-sulfinate are two strong nucleophiles which could add to quinone ring via a nucleophilic addition reaction. Electrochemical methods are considered as green methods for the synthesis of organic compounds and therefore the second part of this study was aimed at the preparation of a few novel CA analogues with a benzenesulfonyl or toluene-4-sulfonyl group attached to their catechol ring using electrochemical method (22-23).

*Chemistry*

**Figure 3:**
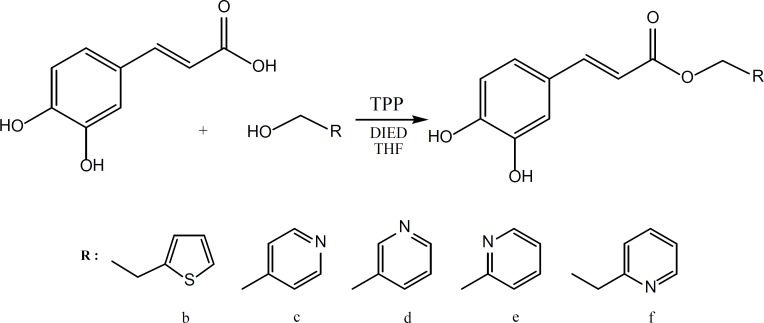
Synthesis of caffeic acid esters (2-6) using Mitsunobu reaction

Caffeic acid and alcohols b-f were condensed in equimolar ratio in presence of triphenylphosphine (TPP) and diisopropylazodicarboxylate (DIAD) in dry tetrahydrofurane (THF) as solvent at room temperature. (Figure 3).

Liquid-liquid extraction was used to extract the crude product and final purification was performed by silicagel column chromatography using chloroform-methanol or ethylacetate-petrolium ether as the eluent solvent. The synthesized compounds were characterized using IR, 1HNMR, 13C-NMR spectroscopies, ESI-Mass spectrometry and elemental analysis.

**Figure 4:**
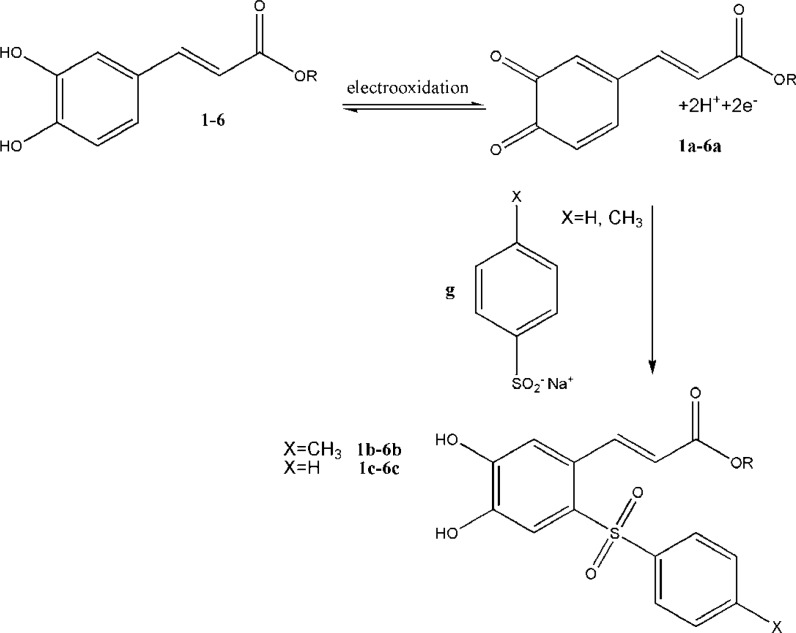
Electroxidation of CA esters (1-6) and reaction with sodium benzenesulfinate or sodium toluene-4-sulfinate

All the heterocyclic esters of caffeic acids (2-6) and CAPE (1) were subjected to electrochemical oxidation in the presence of sodiumbenzenesulfinate or sodium benzenesulfinate or sodium toluene-4-sulfinate as nuleophiles (Figure 4). 

**Figure 5:**
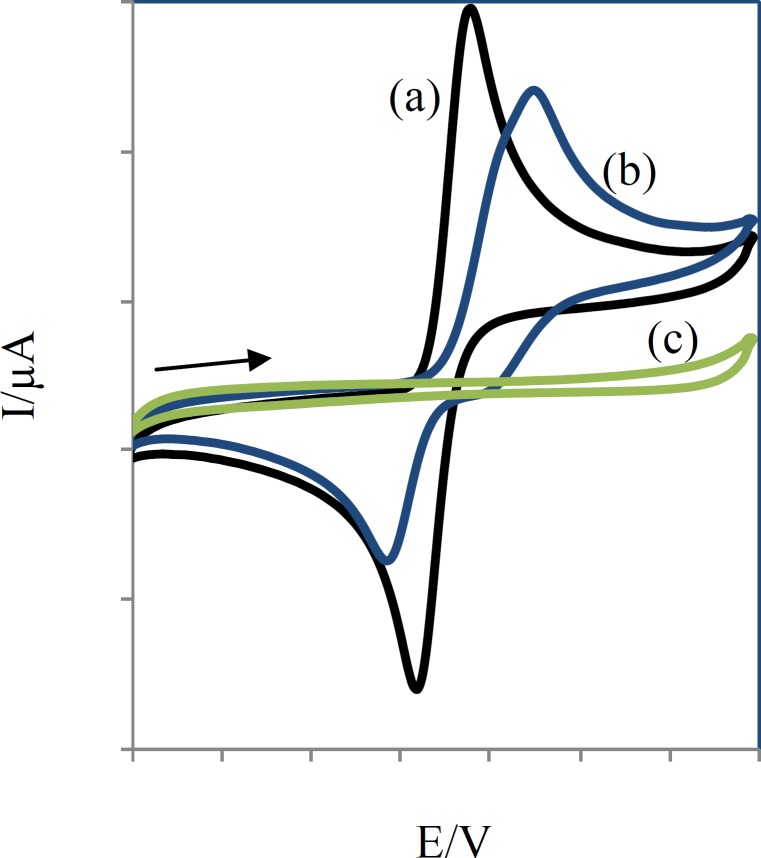
Comparative cyclic voltammograms of 0.25 mM compound 2 without (a), with (b) 0.25 mM sodium toluene-4-sulfinate and 0.25 mM sodium toluene-4-sulfinate (c) at a glassy-carbon electrode (S = π mm2) in aqueous solution (H2O:AN, 80:20), containing acetate buffer (pH =7, c = 0.2 M). Scan rate: 50 mV/s

Compounds (1-6) were thus electro-oxidized to their corresponding quinonic forms (1a-6a) on the surface of glassy carbon electrode. When the electro-oxidation process takes place in the presence of sodium benzenesulfinate or toluene-4-sulfinate, the sulfinate group can attack the quinonic ring in a Michael type addition reaction. The quinonic ring could then be reduced electrochemically using a cyclic voltammetric process to generate the reduced form of CA esters. The electrochemical behavior of CA esters (1-6) were studied in the absence and presence of the nucleophiles sodium benzenesulfinate and sodium toluene-4- sulfinate (Figure 5).

Voltammograms of 2 in the absence and presence of sodium toluene-4-sulfinate (Figure 5) show anodic peaks at 359 mV and 449 mV and cathodic peaks at 239 mV and 169 mV, respectively. 

In this condition, part (c) of Figure 5 is related to the electrochemical oxidation of sodium toluene-4-sulfinate. Anodic peaks indicate the oxidation of 3a-e to their corresponding orthobenzoquinones which are reduced back to their initial catechols form by reversing the voltage. The ratio of the oxidation and reduction current amplitudes for all in the oxidation and reduction process were equal to unity. This phenomenon shows the stabilization of o-benzoquinone produced on the surface of electrode. Side reactions, such as dimerization or hydroxylation of (compounds 1-6) are too slow to be observed on the time scale of cyclic voltammograms. 

When compounds sodium benzenesulfinate or sodium toluene-4-sulfinate were added to the solution containing (compounds 1-6), the anodic peaks shifted positively in all cases (part (b) of Figure 5) and the cathodic peak currents decreased. The positive shift of anodic potential could be related to the formation of a thin film on the surface of the electrode. The formation of toluene-4-sulfinate adducts (1b-6b) and benzenesulfinate adducts (1c-6c) was confirmed by thin layer chromatography (TLC) and electrospray ionization mass spectrometry (ESI-MS). 

*Pharmacology*

*Cell Culture*

Human ovarian adenocarcinoma cell line SK-OV-3 (ATCC no. HTB-77), human cervix adenocarcinoma cell line HeLa (ATCC no.CCL-2), and human colon adenocarcinoma HT-29 (ATCC no. HTB-38) were obtained from American Type Culture Collection. Cells were grown on 75 cm^2^ cell culture flasks with EMEM (Eagle’s minimum essential medium), supplemented with 10% fetal bovine serum, and 1% penicillin/streptomycin solution (10,000 units of penicillin and 10 mg of streptomycin in 0.9% NaCl) in a humidified atmosphere of 5% CO2, 95% air at 37 ºC.

**Figure 6:**
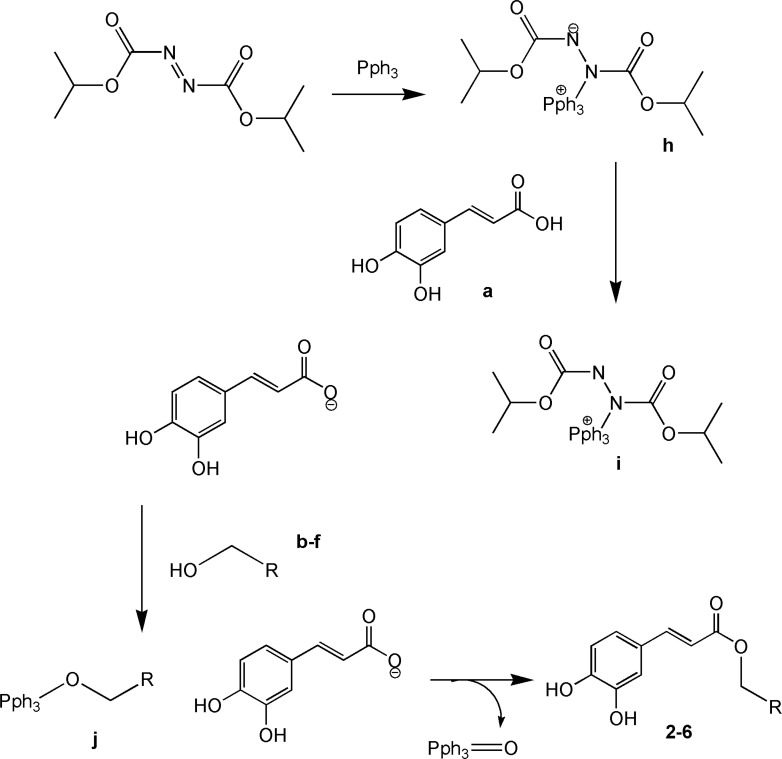
Mechanism of Mitsunobu reaction

*Cell proliferation assay*

Cell proliferation assay was carried out using CellTiter 96 aqueous one solution cell proliferation assay kit (Promega, USA). Briefly, upon reaching about 75-80% confluency, 5000 cells/well were plated in 96-well microplate in 100 μL media. After seeding for 72 h, the cells were treated with 50 μM compound in triplicate. Doxorubicin (10 μM) was used as the positive control. At the end of the sample exposure period (72 h), 20 μL CellTiter 96 aqueous solution was added. The plate was returned to the incubator for 1 h in a humidified atmosphere at 37 ºC. The absorbance of the formazan product was measured at 490 nm using microplate reader. The blank control was recorded by measuring the absorbance at 490 nm with wells containing medium mixed with CellTiter 96 aqueous solution but no cells. Results were expressed as the percentage of the control (without compound set at 100%). The results are shown as the percentage of the control DMSO that has no compound (set at 0%). All the experiments were performed in triplicate.

## Result and Discussion

*Chemistry*

Esters are traditionally produced by condensing carboxylic acids and alcohols in the presence of an acid catalyst. The esterification reaction is both slow and reversible. There are many ways to drive the equilibrium toward the ester product: a: addition of an excess of one of the reactants (usually the alcohol), b: removal of the ester or the water by distillation, c: removal of water by azeotropic distillation and d: removal of water by use of dehydrating agent or molecular sieve.

When carboxylic acids and/or alcohols have other functional groups which are sensitive to the estrification conditions, unconventional methods should be employed to circumvent the vulnerability of these functional groups. Phenolic hydroxyl groups are among the interfering functional groups with the ordinary process of esterification. 

Caffeic acid is a polyphenolic *α-β *unsaturated carboxylic acid with more issues in esterification reaction than just being a simple phenol. Therefore special conditions should be used for its esterification. In the present study we compared the following esterification methods for caffeic acid:

I. Acid catalyzed esterification with H_2_SO_4_.

II. Microwave assisted esterification in presence of a protic acid.

III. Ultrasound assisted esterification in presence of a protic acid.

IV. Estrification with the aid of thionyl chloride.

V. Esterification with the aid of dicyclohexyl dicarbondiimide(DCC).

VI. Wittig reaction between 3, 4-dihydroxy benzaldehyde and phosphonium chloride salt of alcohol.

VII. Knoevenagel reaction.

VIII. Esterification after protection of phenolic hydroxyl groups by BBr3 and tetrahydropyrane.

IX. Mitsunobu reaction.

None of the above mentioned (except Mitsunobu reaction) led to the satisfactory results either because the reaction did not give the desired product at all (I, II, III, IV, V, VIII) or due to the formation of a great number of byproducts which made the purification of the desired product impossible (VI, VII, IX).

Mitsunobu reaction on the other hand was successful in giving the desired products for the reaction of caffeic acid with the alcohols b-f.

Mitsunobu reaction is a method for converting molecules that bear poor leaving group into a derivative that can react via SN2 displacement, even when the nucleophile is as weak as a carboxylate anion. The process involves reaction of diisopropylazodicarboxylate (DIAD) with triphenylphosphine to form h. This dipolar ion reacts with RCOOH, which is present in the initial reaction to give i. The alcohol is then added to this phosphonium salt, and the subsequent reaction generates alkoxyphosphonium salt j. The RCOO – group displaces triphenylphosphin oxide to give the desired product.

It is generally accepted that the acids with a pKa< 11 will be successful in Mitsunobu reaction. In light of this fact, it is not surprising that the acidic phenolic hydroxyl groups of caffeic acid would not interfere with this reaction. The yields for the Mitsunobu reaction conducted for caffeic acid reaction with alcohols b-f are presented in Table 1.

**Table 1:** The yields and mp for Mitsunobu reaction of caffeic acid with alcohols 2a-e

**Alcohol**	**yield**	**mp°C **
b	10%	138.5-140
c	25%	167-169
d	15%	70-72
e	18%	154-157
f	20%	200-203

The purification of esters 2-6-e was performed on silica gel. This is more convenient than the use of Sephadex LH-20 which has been used by Appendino *et al*. in their report (21).

In general due to the mild conditions and the possibility of purification polyphenolic esters on silica gel column, the Mitsunobu reaction is particulary suitable for the synthesis of polyphenolic compound such as caffeic acid esters, a group of compound with interesting biological activities. 

*Cytotoxic activity*

The results of cytotoxic activity were summarized as IC_50_ (μmolar) of compounds in Table 2.

**Table 2:** Cytotoxic activity (IC_50_, μm) of compounds 1-6, 1b-6b and 1c-6c

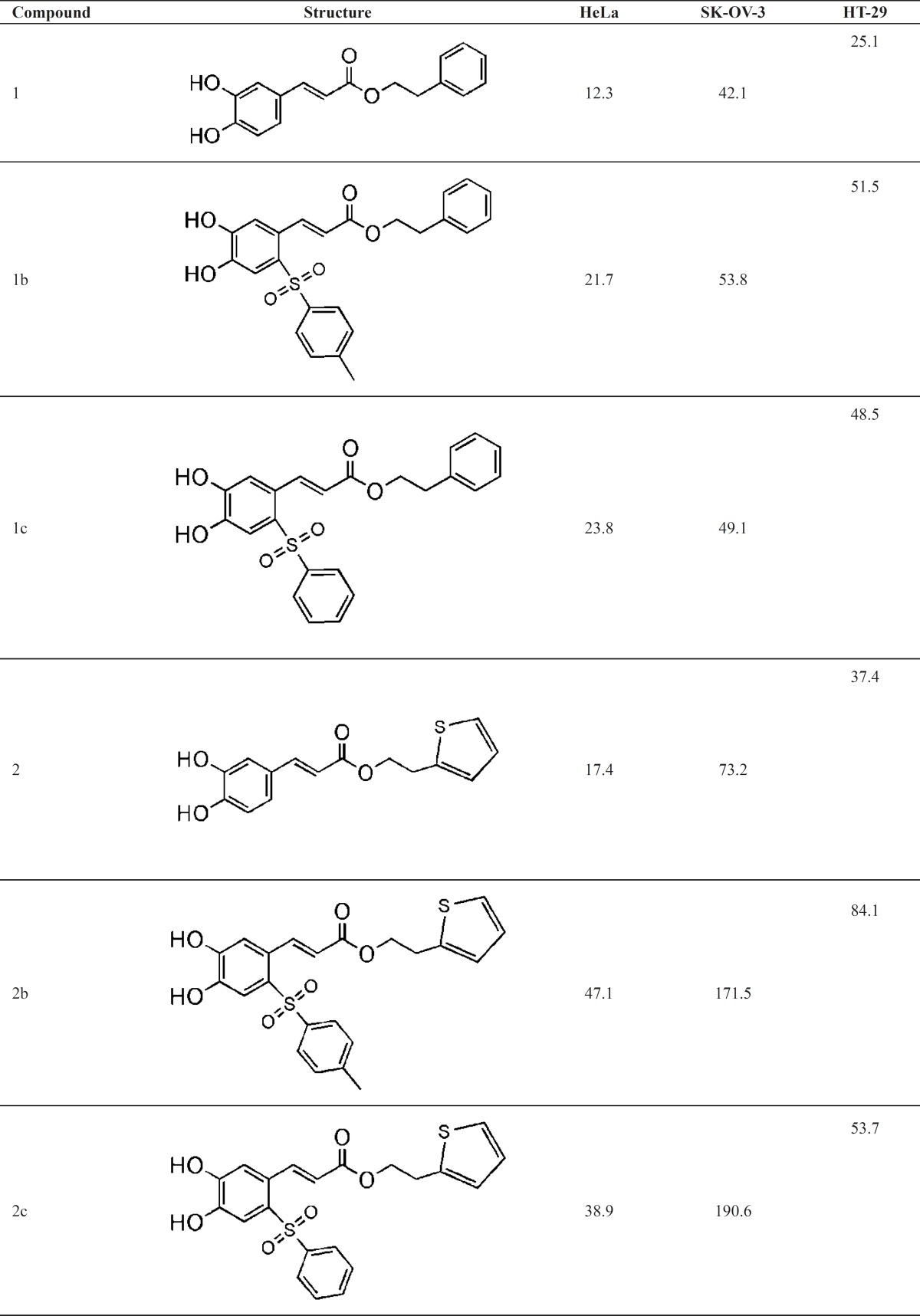

In general all compounds had good inhibitory activity (IC_50_=10-200 μM) against HeLa and HT-29 but they did not show significant inhibitory activity (IC_50_ > 200 μM) against SK-OV-3. All these esters except 6, 6b, and 6c showed the strongest activity toward colon HT-29 cell line. All the benzenesulfonyl (1b-6b) and toluene-4-sulfonyl (1b-6b) derivatives showed decreased activity. It could be speculated that the arylsulfonyl group causes an electron deficiency and/or steric hindrance on catechol ring and therefore interfere with the normal oxidation/reduction of catechol ring. Comparing the IC_50_ values of structures 1-6 indicates that thiophen is more successful than pyridine.

2-(2-pyridyle) ethyl analogues of CAPE (5, 5b, 5c) have stronger cytotoxicity than 2-pyridyle methyl analogues (6, 6b, 6c). This is in agreement with the previous finding for CAPE which shows that phenethyl esters is more potent than phenmethyl ester of caffeic acid.

*QSAR study*

In order to evaluate the effects of the structural parameters of the investigated compounds 1-6, 1b-6b and 1c-6c on their cytotoxic activities, Quantitative-Structure Activity Relationship (QSAR) analysis with different types of molecular descriptors was performed. 

Several physicochemical descriptors such as hydrophobicity, topological indices, electronic parameters and steric factors are usually used in QSAR studies in order to find the effects of different structural properties on the biological activity of compound of interest. Since the calculated values of some electronic descriptors depend on the three-dimensional molecular geometry, the optimum 3-D geometry of the molecules were obtained by Hyperchem software (Hypercube Inc, USA), using AM1 semi-empirical method. The resulting structures were used to calculate constitutional, functional, geometrical and topological descriptors by Dragon software. Meanwhile some electronic descriptors such as frontier molecular orbital (HOMO, LUMO), dipole moment and partial charges were calculated by the Hyperchem software. Activity data were converted to logarithmic scale (*i.e*. pIC_50_ for cytotoxicity in different cell lines). For each set of descriptors, the best multi-linear regression equations were obtained by the stepwise selection methods of multiple linear regression (MLR) subroutine of SPSS software. The correlation coefficient (r^2^), standard error of regression (SE), correlation coefficient for cross-validation significance (q^2^), root mean square error (RMS) and significant level (p-value) were employed to judge the validity of regression equation. As colinearity degrades the performances of the MLR-based QSAR equation, at the first, correlation analysis was performed to detect the co-linear descriptors (24). Thus, the correlation of descriptors with each other and with activity data was examined and among the co-linear descriptors one of them that represented the highest correlation with activity was retained and the rest were omitted. The resulted correlation matrix is represented in Table 3 and 4 for the remaining descriptors.

**Table 3:** Correlation coefficient (R^2^) matrix for some of descriptors used for HeLa

	**ASP**	**GATS3e**
ASP	1	0.234
GATS3e		1

In the first step, it was tried to find an appropriate model for cytotoxic activity in HeLa cell line. The obtained equation is shown by Equation 1:

pIC_50_= 0.416 ± (0.021) ASP + 0.129 ± (0.054) GATS3e + 3.574 ± (0.098) (Equation 1)

n = 18 r^2^ = 0.968 RMScv = 0.019 SE = 0.015 q2 = 0.941 F = 226

In this equation, the values in the parenthesis represent the standard deviation of the coefficients.

This model contains asphericity (ASP), Geary autocorrelation of lag 3 weighted by Sanderson electronegativity (GATS3e).

The next equation was obtained for cytotoxicity against Ht_29 cell line: 

pIC_50_= 0.393 ± (0.016) GATS1e -0.081 ± (0.023) GATS3v + 4.015 ± (0.052) (Equation 2) 

n = 18 r^2^ = 0.976 RMscv = 0.013 SE = 0.011 q2 = 0.966 F = 311

Equation 2 includes Geary autocorrelation of lag 1 weighted by Sanderson electronegativity (GATS1e), Geary autocorrelation of lag 3 weighted by van der Waals volume (GATS3v). The values of the descriptors used by Equations1 and 2 together with the predicted -log IC_50_ (pIC_50_) are listed in Table 3, 4.

**Table 4:** Correlation coefficient (R_2_) matrix for some of descriptors used for Ht-29

	**GATS1e**	**GATS3v**
GATS1e	1	0.248
GATS3v		1

**Table 5:** Data of the selected descriptors used in this study and predicted values of pIC_50 _(HeLa).

**Compound**	**ASP**	**GATS3e**	**Predicted pIC** **50** **(HeLa)**
1	0.600	1.802	4.056
1b	0.239	1.784	3.904
1c	0.238	1.788	3.904
2	0.628	1.971	4.090
2b	0.274	1.891	3.932
2c	0.308	1.902	3.947
3	0.662	1.817	4.084
3b	0.224	1.791	3.898
3c	0.279	1.805	3.923
4	0.659	1.799	4.080
4b	0.227	1.779	3.898
4c	0.282	1.793	3.923
5	0.559	1.996	4.064
5b	0.260	1.912	3.929
5c	0.258	1.922	3.929
6	0.657	1.823	4.082
6b	0.247	1.794	3.908
6c	0.297	1.808	3.931

**Figure 7:**
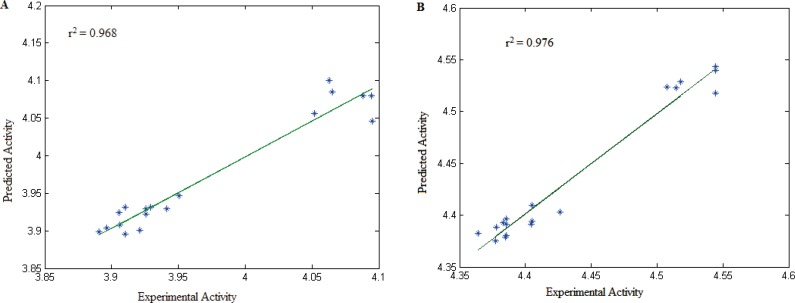
Plots of cross-validated predicted values of activity by MLR against the experimental values for different cell lines: (A) HeLa and (B) HT-29

The correlation between the observed and predicted activities of all the compounds using Equation 1 and Equation 2 is represented graphically in Figure 7A and Figure 7B.

These two equations show that spatial and topological descriptors play important role in cytotoxicity of these compounds. The autocorrelation vectors represent the degree of similarity between molecules. Therefore, cytotoxic activity of these series of compounds is mainly dependant on molecular shape of structure.

**Table 6:** Data of the selected descriptors used in this study and predicted values of pIC_50_ (HT-29)

715 **Compound **	**GATS1e **	**GATS3v **	**Predicted pIC** **50** **(Ht-29) **
1	1.746	2.221	4.521
1b	1.381	2.244	4.376
1c	1.390	2.146	4.387
2	1.773	2.274	4.528
2b	1.400	2.283	4.380
2c	1.412	2.180	4.393
3	1.803	2.446	4.525
3b	1.427	2.394	4.382
3c	1.441	2.293	4.396
4	1.803	2.506	4.521
4b	1.427	2.431	4.379
4c	1.441	2.332	4.392
5	1.780	2.099	4.545
5b	1.416	2.169	4.396
5c	1.427	2.060	4.409
6	1.803	2.255	4.541
6b	1.427	2.276	4.391
6c	1.441	2.166	4.406

## Conclusion

A few heterocyclic esters of caffeic acid were synthesized using Mitsunobu reaction. Other methods of esterification failed to give the desired compounds possibly due to the sensitive nature of catechol ring in the structure of cafeic acid. The esters were further modified by attaching benzensulfonyl or toluene-4-sulfonyl group to their catechol ring using cyclic votammetry method.

Evaluation of cytotoxic activity for the compounds indicates that the compounds are more active against HeLa cancer cell line compared to SK-OV-3 and HT-29.

QSAR analysis reveals that the cytotoxic activity is mainly under the influence of molecular shape of these compounds.

Incorporation of arylsulfonyl groups diminishes the activity, perhaps due to the electron withdrawing and/or steric effect.

## Experimental

*General method*

Chemicals and all solvents used in this study were purchased from Merck AG and Aldrich Chemicals. CAPE (1) was prepared using wittig reaction according to literature report (12). Melting points were determined on a Kofler hot stage apparatus. The IR spectra were obtained on a Shimadzu 470 spectrophotometer (potassium bromide disks). 1H NMR spectra were recorded on a Varian unity 500 spectrometer and chemical shifts (d) are reported in parts per million (ppm) relative to tetramethylsilane (TMS) as an internal standard. Elemental analyses were carried out on a Costec rapid elemental analyzer Model 4010 (GmbH-Germany) for C, H, N and S, and the results are within ± 0.4% of the theoretical values. Merck silica gel 60 F254 plates were used for analytical TLC; column chromatography was performed on Merck silica gel (70–230 mesh).

The electrospray mass spectra (ESI) were recorded on an Agilent 4610 triple quadrupole mass spectrometer.

Cyclic voltmmetry was performed, using Metrom computerized voltammetric analyzer model 746 VA Trace Analyzer/747 VA Stand. Controlled-potental columetry and preparative electrolysis were performed using BHP2050 potentiostat/galvanostat. The working electrode used in the voltammetry studies was a glassy carbon disc (1.8 mm diameter). The potential were measured versus the Ag/AgCl/KCl (3M) as a reference electrode and platinum wire was used as the counterelectrdoe. 

In macroscale electrolysis, four carbon rods (8 mm diameter and 5 cm length) were used as working electrodes.

*General procedure for the synthesis of 2-6*

To a solution of alcohols b-f (2.5 mmole) and caffeic acid (2.5mmole) in dry THF under N_2_ atmosphere, were added TPP (2.3 mmole) and DIAD (2.3mmole) at 0 °C. The reaction mixture was stirred at room temperature for 2 days. Then, the reaction was worked up by removal of the solvent, redissolving the residue in ethyl acetate and washing with 1N NaHCO_3_ (ca. 50 mL/250mg of alcohol). The product obtained after evaporation of ethylacetate was purified by silica gel column chromatography, eluting with ethyl acetate-petrolium ether (20:80 for 3-6) or chloroform-methanol (95:5 for 4).

*(E)-2-(thiophen-2-yl) ethyl 3-(3,4-dihydroxyphenyl)acrylate (2)*

IR, *v*/cm^-1^: 3465, 3224, 2924, 2854, 1671, 1603; 1H NMR (DMSO-*d6*), *δ*: 9.5 (bs, 2H, OHs), 7.48 (d, *J*=16Hz, 1H, olefinic H), 7.35 (d, *J*=5 Hz, 1H, H-5 of thiophene ring), 7.04 (s, 1H, H-2 of cathecol ring), 6.99 (d, *J*=8 Hz, 1H, H-6 of chatecol ring),6.96 (m, 2H, H-3 and H-4 of thiophene ring), 6.75(d, *J*=8Hz, 1H, H-5 of cathecol ring), 6.25 (d, *J*=16 Hz, 1H, olefinic H), 4.30 (t, *J*=6.5 Hz, 2H, CH_2_-2), 3.17 (t, *J*=6.5 Hz, 2H, CH2-1); 13C NMR (DMSO-*d6*), δ: 166.4, 148.5, 145.5, 145.3, 140, 126.9, 125.7, 125.4, 124.4, 121.4, 115.7, 114.8, 113.7, 64, 21.9. MS, *m/z *(Ir/%): 291.06 (M+). Anal. Calcd. For C_15_H_14_O_4_S: C, 62.05; H, 4.86. Found: C, 62.27; H, 4.72. 

*(E)-pyridin-4-ylmethyl 3-(3, 4-dihydroxyphenyl)acrylate (3)*

IR, *v*/cm^-1^: 3437, 3033, 2924, 1711, 1635, 1602; 1H NMR (DMSO-*d6*), *δ*: 9.66 (s, 1H, OH), 9.17 (s, 1H, OH), 8.56 (d, *J*=6Hz, 2H, H-2 and H-6 of pyridine ring), 7.57 (d, *J*=15.5Hz, 1H, olefinic H), 7.38 (d, *J*=6Hz, 2H, H-3 and H-5 of pyridine ring), 7.08 (d, *J*=2Hz, 1H, H-2 of cathecol), 7.04 (dd, *J*=8Hz, 2Hz, 1H, H-6 of cathecol), 6.76 (d, *J*=8Hz, 1H, H-5 of cathecol ring), 6.37(d, *J*=15.5Hz, olefinic H), 5.24 (s, 2H); 

^13^C NMR (DMSO-*d6*), δ: 166.2, 149.7, 149.2, 148.6, 146, 145.5, 125.4, 121.8, 121.6, 121, 115.7, 114.9, 113.2, 63.5, 61.4. MS, *m/z *(Ir/%): 272.08 (M^+^). Anal. Calcd. For C_15_H_13_NO_4_: C, 66.41; H, 4.83; N, 5.16. Found: C, 66.58; H, 5.02; N, 5.31.

*(E)-pyridin-3-ylmethyl 3-(3,4-dihydroxyphenyl)acrylate (4)*

IR, *v*/cm^-1^: 3441, 3060, 2928, 1713, 1632, 1602; 1H NMR (DMSO-*d6*), *δ*: 9.5(bs, 2H, OHs), 8.63 (s, 1H, H-2 of pyridine ring), 8.54 (d, *J*=4.5Hz, 1H, H-6 of pyridine ring), 7.83 (d, *J*=7.5Hz, 1H, H-4 of pyridine ring), 7.53 (d, *J*=15.5Hz, 1H, olefinic H), 7.41 (dd, *J*=7.5Hz, 4.5Hz, 1H, H-5 of pyridine ring), 7.06 (d, *J*=2Hz, 1H, H-2 of cathecol ring), 7.01 (dd, *J*=8.5Hz, 2Hz, 1H, H-6 of cathecol ring), 6.75 (d, *J=*8.5Hz, 1H, H-5 of cathecol ring), 6.32 (d, *J*=15.5Hz, 1H, olefinic H), 5.23 (s, 2H, CH_2_); 13C NMR (DMSO-*d6*), δ: 166.3, 149.3, 148.5, 148.1, 145.8, 136, 132.1, 125.4, 123.6, 121.5, 115.7, 114.9, 113.4, 63, 60.6. MS, *m/z *(Ir/%): 272.08 (M+). Anal. Calcd. For C15H13NO4: C, 66.41; H, 4.83; N, 5.16. Found: C, 66.79; H, 4.62; N, 5.44.

*(E)-2-(pyridin-2-yl) ethyl 3-(3,4-dihydroxyphenyl)acrylate (5)*

IR, *v*/cm^-1^: 3449, 3214, 2897, 1689, 1637, 1599; 1H NMR (DMSO-*d6*), *δ*: 9.4(bs, 2H, OHs), 8.5 (d, *J*=5 Hz, 1H, H-6 of pyridine ring), 7.71 (t, *J*=7.5 Hz, 1H, H-4 of pyridine ring), 7.42 (d, *J*=16 Hz, 1H, olefinic H), 7.32 (d, *J*=7.5Hz, 1H, H-3 of pyridine ring), 7.23 (t, *J*=5Hz, 1H, H-5 of pyridine ring), 7.01 (s, 1H, H-2 of cathecol ring), 6.97 (d, *J*=8Hz, 1H, H-5 of cathecol ring), 6.74 (dd, *J*=8Hz, 2Hz, 1H, H-6 of cathecol ring), 6.2 (dd, *J*=16Hz, 2Hz, 1H, olefinic H), 4.47 (t, *J*=6.5Hz, 2H, CH_2_-2), 3.09 (t, *J*=6.5Hz, 2H, CH_2_-1); 13C NMR (DMSO-*d6*), δ: 166.5, 157.9, 149.1, 148.4, 145.5, 145.2, 136.5, 125.4, 123.4, 121.7, 121.4, 115.7, 114.8, 113.8, 62.9, 36.6. MS, *m/z *(Ir/%): 286.10 (M^+^). Anal. Calcd. For C_16_H_15_NO_4_: C, 67.36; H, 5.30; N, 4.91. Found: C, 67.01; H, 5.42; N, 4.73.

*(E)-pyridin-2-ylmethyl 3-(3,4-dihydroxyphenyl)acrylate (6)*

IR, *v*/cm^-1^: 3476, 3056, 2953, 2563, 1693, 1600; 1H NMR (DMSO-*d6*), *δ*: 9.63 (s, 1H, OH), 8.55 (d, *J*=4 Hz, 1H, H-6 of pyridine ring), 7.82 (t, *J*=8 Hz, 1H, H-4 of pyridine ring), 7.55 (d, *J*=16 Hz, 1H, olefinic H), 7.43 (d, *J*=7.5 Hz, 1H, H-3 of pyridine ring), 7.33 (dd, *J*=8 Hz, 4Hz, 1H, H-5 of pyridine ring), 7.07 (s, 1H, H-2 of cathecol ring), 7.03 (d, *J*=8 Hz, 1H, H-6 of cathecol ring), 6.76 (d, *J*=8 Hz, 1H, H-5 of cathecol ring), 6.37 (d, *J*=16 Hz, 1H, olefinic H), 5.25 (s, 2H, CH_2_); 13C NMR (DMSO-*d6*), δ: 166.2, 155.9, 149.1, 148.5, 145.8, 145.6, 136.9, 125.4, 122.9, 121.5, 115.7, 114.9, 113.5, 65.9, 63.9. MS, *m/z *(Ir/%): 272.08 (M^+^). Anal. Calcd. For C_15_H_13_NO_4_: C, 66.41; H, 4.83; N, 5.16. Found: C, 66.64; H, 4.52; N, 5.31.

*General procedure for electroorganic synthesis of 1b-6b and 1c-6c*

The mixture of water-acetonitrile (AN) (80:20), containing phosphate buffer (pH= 7.0, C=0.2M), was preelectrolyzed at 0.3V.

Equimolar amounts of caffeic acid eters and sodium benzenesulfinate or (sodium toluene-4-sulfinate) were added to the cell with 4 graphit rodes as working electrode and Pt electrode as counter electrode.

The potentials of working electrode were measured versus the Ag/AgCl/KCl as a reference electrode. The electrolysis was interrupted many times, when the current reached to 5% of the starting value, to wash the anodic electrode with acetone to reactivate it. The precipitated products were filtered off and washed with water/acetone mixture.

*Compounds 1b-6b and 1c-6c*

Compounds were characterized by ESI mass spectrometry and all of the compounds were confirmed by observing their pseudomolecular ion as hydrogen adduct.
